# A life cycle assessment of reprocessing face masks during the Covid-19 pandemic

**DOI:** 10.1038/s41598-021-97188-5

**Published:** 2021-09-03

**Authors:** Bart van Straten, S. Ligtelijn, L. Droog, E. Putman, J. Dankelman, N. H. Sperna Weiland, T. Horeman

**Affiliations:** 1 Department of BioMechanical Engineering, Delft University of Technology, Mekelweg 2, Building 34, 2628 CD Delft, The Netherlands; 2 Industrial Ecology, Delft University of Technology and Leiden University, Delft, The Netherlands; 3 VWS, Ministry of Health, Welfare and Sport (VWS), The Hague, The Netherlands; 4 Amsterdam University Medical Center, Amsterdam, The Netherlands

**Keywords:** Climate-change ecology, Engineering, Biomedical engineering

## Abstract

The Covid-19 pandemic led to threatening shortages in healthcare of medical products such as face masks. Due to this major impact on our healthcare society an initiative was conducted between March and July 2020 for reprocessing of face masks from 19 different hospitals. This exceptional opportunity was used to study the costs impact and the carbon footprint of reprocessed face masks relative to new disposable face masks. The aim of this study is to conduct a Life Cycle Assessment (LCA) to assess and compare the climate change impact of disposed versus reprocessed face masks. In total 18.166 high quality medical FFP2 face masks were reprocessed through steam sterilization between March and July 2020. Greenhouse gas emissions during production, transport, sterilization and end-of-life processes were assessed. The background life cycle inventory data were retrieved from the ecoinvent database. The life cycle impact assessment method ReCiPe was used to translate emissions into climate change impact. The cost analysis is based on actual sterilization as well as associated costs compared to the prices of new disposable face masks. A Monte Carlo sampling was used to propagate the uncertainty of different inputs to the LCA results. The carbon footprint appears to be 58% lower for face masks which were reused for five times compared to new face masks which were used for one time only. The sensitivity analysis indicated that the loading capacity of the autoclave and rejection rate of face masks has a large influence on the carbon footprint. The estimated cost price of a reprocessed mask was €1.40 against €1.55. The Life Cycle Assessment demonstrates that reprocessed FFP2 face masks from a circular economy perspective have a lower climate change impact on the carbon footprint than new face masks. For policymakers it is important to realize that the carbon footprint of medical products such as face masks may be reduced by means of circular economy strategies. This study demonstrated a lower climate change impact and lower costs when reprocessing and reusing disposable face masks for five times. Therefore, this study may serve as an inspiration for investigating reprocessing of other medical products that may become scarce. Finally, this study advocates that circular design engineering principles should be taken into account when designing medical devices. This will lead to more sustainable products that have a lower carbon footprint and may be manufactured at lower costs.

## Introduction

According to the European Commission, the European recovery plan for the economy after Covid-19 aims to make the European economy more circular and more sustainable. The Green Deal on Sustainable Healthcare^1^, as set out in The Netherlands, consists of a formal contract signed by hospitals, government organisations, industrials and universities, and will be used in the European's recovery strategy by stimulating a circular economy^2^ by building a more resilient European Union. One of the goals as seen from the Dutch Green Deal on Sustainable Healthcare is to reduce waste. By 2030, the CO_2_ emissions from healthcare should be reduced by 49% compared to the 1990 levels, and by 2050 realise a climate neutral situation^1^.

Hospital waste production in high income countries varies between 1.7 and 8.4 kg per bed per day depending on hospital size and activities^3^. For hospitals in Europe ranging from 1.7 kg in the Netherlands to 3.6 kg in Germany, between 3.6 and 4.0 in Middle East countries such as Kuwait^4^. and in the US these numbers are rising to 8.4 kg^5^. In total 5.9 million tons of medical waste is disposed in the USA by hospitals annually and healthcare produces 8% of the total CO_2_ emissions in the US^6^. Subsequently, severe health risks associated with medical waste disposal by hospitals have been reported^7^.

The Covid-19 pandemic led to severe shortages of medical products in particular with personal protective equipment (PPE)^8^. These local shortages of PPE included face masks, aprons and isolation gowns. This period led to an emergency scenario in which reprocessing was devised as an alternative. This resulted in situations in which either no care could be given or in situations where health care professionals were not fully protected. The authorities decided to temporarily exempt these medical products from CE registration^9^. Meaning that manufacturers and suppliers were able to supply non-CE-marked medical equipment, such as face masks, at the explicit request of hospitals or other healthcare institutions, when shortages occurred as a result of the coronavirus.

Upon request of several hospitals, a variety of methods for reprocessing were investigated of single use face masks in the period starting at 17 March 2020^10^. The quality of reprocessed as well as new face masks were tested with a custom test set-up which was built to measure the filter penetration of particles with different size and pressure drop over the face masks. With this system the filter capacity and filter material pressure drop of, commonly used, sterilized masks were evaluated between 17 March and 1 July 2020^11^.

In total 18,166 FFP2 face masks were steam sterilized at 121 °C and 88 different masks brands were evaluated, showing that the Particle Filtration Efficiency (PFE) in the particles range of 0.3, 0.5 and 5 microns did not change significantly for the commonly used 3M 1862+ masks with and without a valve. The study indicates that for reuse up to five times after multiple heat sterilization procedures the PFE remained, with an averaged minimum of 96,8% for the smallest particle size, well above the FFP2 standard of 94% for particles larger than 0,3 micron^10,12^. In addition, the pressure drop measured for 3M 1862+ masks that were used and reprocessed up to 3 times showed that the pressure drop remained well below the 0,7 Mbar standard as defined in the EN-149 with values around 0.2 Mbar^11,12^. As leakage is a very relevant aspect, all masks seals were visually and tactilely inspected for damages and changes in elasticity before the masks were placed on the face and used^11^. Reprocessing by means of steam sterilization of disposable face masks at 121 °C showed acceptable PFE results, maintaining its filtration material quality, and can be done if the fit does not change^10^. Our suggestion that 3M 1862+ masks can be reprocessed was later confirmed in a technical bulletin by the manufacturer of the masks, 3M^13^ and by the National Institute for Public Health and the Environment^14^. These studies demonstrated that a circular approach for certain face masks is feasible.

The Corona crisis period appeared to provide a potential motive to investigate the effects of reprocessing medical equipment. Since the circular reprocessing involved steam sterilization, it was of equal importance to determine whether this approach is sustainable. This study was therefore conducted to demonstrate the environmental sustainability by means of a Life Cycle Assessment (LCA) and to investigate economic feasibility. The carbon footprint (expressed in kg CO_2_ eq) and costs of reprocessed face masks and new ones will be studied from a circular economy perspective.

Several studies assessed the impact of facemasks or other PPE from different perspectives. Allisson et al.^15^, performed a Life Cycle Assessment as well as a cost comparison to demonstrate that reusable face masks have a lower environmental and economic impact when compared to single-use face masks. Kumar et al.^16^ conducted a Life Cycle Assessment of Personal Protective Equipment (PPE) where cycles during end-of-life PPE to landfill and incineration were investigated. Schmutz et al.^17^ investigated the ecological factors by comparing surgical masks with cotton masks. However, the perspective of our study is to identify the differences in climate change impact when reusing the same single-use face mask five times.

The aim of this study is to conduct a Life Cycle Assessment (LCA) to assess and compare the climate change impact of disposed versus reprocessed face masks. The following research questions were formulated:

1. What is the climate change impact of reprocessed versus disposable FFP2 face masks?
2. What are the financial differences of reprocessed versus disposable face masks?

## Methods

### Scope

We compared disposable face masks that were used once with face masks that were sterilized and used five more times (six times in total). Sterilisation and PFE test data of the Aura 1862+ (3M, Saint Paul, Minnesota, USA) face mask indicate that this type of face mask shows good performance after multiple sterilisation cycles^10–12^. In a previous pilot study, the company CSA Services (Utrecht, the Netherlands), a sterilization facility for cleaning, disinfection and sterilization of medical instruments, was rebuild to process FFP2 face masks. In total, 18,166 single use FFP2 masks were sterilised after use in a medical autoclave. As the majority (n = 7993) were Aura 1862+ (3M, Saint Paul, Minnesota, USA), this particular type of face mask was chosen for the LCA.

The total weight of the face masks and packaging together during end-of-life consists of incineration for the face masks (97%) and landfill for the carton box packaging of new face masks (3%). There is no recycling potential used in our model since the materials coming from the operating room and its packaging is commonly disposed as medical waste. In the Netherlands, no energy recovery takes place at the incineration of regulated medical waste. Therefore, no co-function was applicable for the end-of-life scenario.

Recycling is often a multi-functional process that produces two or more goods. To deal with the multi-functionality in the background processes, the cut-off approach was applied to exclude the allocation of the greenhouse gas emissions to additional goods. This means that potential rest materials such as energy gained during incineration are cut-off and that the greenhouse gas emissions are fully allocated to the waste treatment processes itself.

In the LCA, the ‘functional unit’ defines the primary function that is fulfilled by the investigational products and indicates how much of this function is considered^18^. In this study, we pragmatically chose as a definition for the protection of 100 health care workers against airborne viruses, using one FFP2 certified face mask, each during one working shift of an average of 2 h in a hospital in the Netherlands.

Table 1 shows the differences between the two scenarios:

1. 100 masks including packaging, transported from production to the hospital, used and disposed.
2. 100 times use of reprocessed masks. We calculated that 27.1 masks are being produced and transported from production to the hospital. The 27.1 are being reprocessed five times, taking into account that 20% of the batch cannot be reprocessed. Therefore 80% of the batch could be used for reprocessing after each step resulting in: 27.1 (new) + 21.7 (repro 1) + 17.3 (repro 2) + 13.9 (repro 3) + 11.1 (repro 4) + 8.9 (repro 5) = 100 times of use. For each time of reprocessing the batch is transported from the hospital to the (hospital) Central Sterilization Services Department (CSSD) and disposed after five times of reprocessing.

**Table 1:** Comparison between reference flow 1 and 2.

	Times (re)sterilized	Purchased masks	Reprocessed	Disposal	Times protection
Reference flow 1	0	100	0	100.0	100.0
Reference flow 2	0	27.1	27.1	0.0	27.1
	1	0.0	21.7	5.4	48.8
	2	0.0	17.3	4.3	66.1
	3	0.0	13.9	3.5	80.0
	4	0.0	11.1	2.8	91.1
	5	0.0	8.9	2.2	100.0

Combining the functional unit with the two alternative scenarios results in the reference flows for the protection of 100 health care workers against airborne viruses, either using a face mask one single time (100 virgin masks produced for the 1st scenario), or reusing a face mask for five additional times (27.1 virgin masks produced for the 2nd scenario). For both reference flows, only FFP2 certified face masks are considered. For the calculations each mask is used for a single two hours working shift in an average hospital in the Netherlands.

### Life cycle inventory (LCI) analysis

The inventory data includes all phases from production (including material production and part production), transport, sterilisation to end-of-life of the life cycle of the single use and reprocessed face masks. We disassembled one face mask to obtain the weight of each individual component on a precision scale (Fit Evolve, Bangosa Digital, Groningen, the Netherlands) with a calibrated inaccuracy of 1.5%. Component information and materials were obtained from the data fact sheet provided by the manufacturer. We conducted a separate validation experiment to establish the material composition in the filtering fabric (Supplement file).

This LCA with the Aura 3M masks was based on steam sterilization by means of a hospital autoclave and therefore part of this study. Therefore, face masks were placed in a sterilization bag that contained up to five masks. A total of 1000 masks were placed into an autoclave (Getinge, GSS6713H-E, Sweden) per cycle. After sterilization, the masks were transported to the hospital. Masks were reprocessed for a maximum of five times before final disposal^10,11^.

The assessment of climate change impact is done following as closely as possible the internationally accepted Life Cycle Assessment (LCA) method following the ISO 14040 and 14044 standards^19,20^. The LCA examines all the phases of the product's life cycle from raw material extraction to production, packaging, transport, use and reprocessing until final disposal^19^. The LCA was modelled using SimaPro 9.1.0.7 (PRé Sustainability, Amersfoort, The Netherlands). The background life cycle inventory data were retrieved from the ecoinvent database (Ecoinvent version 3.6, Zürich, Switzerland)^21^.

**Figure 1:**
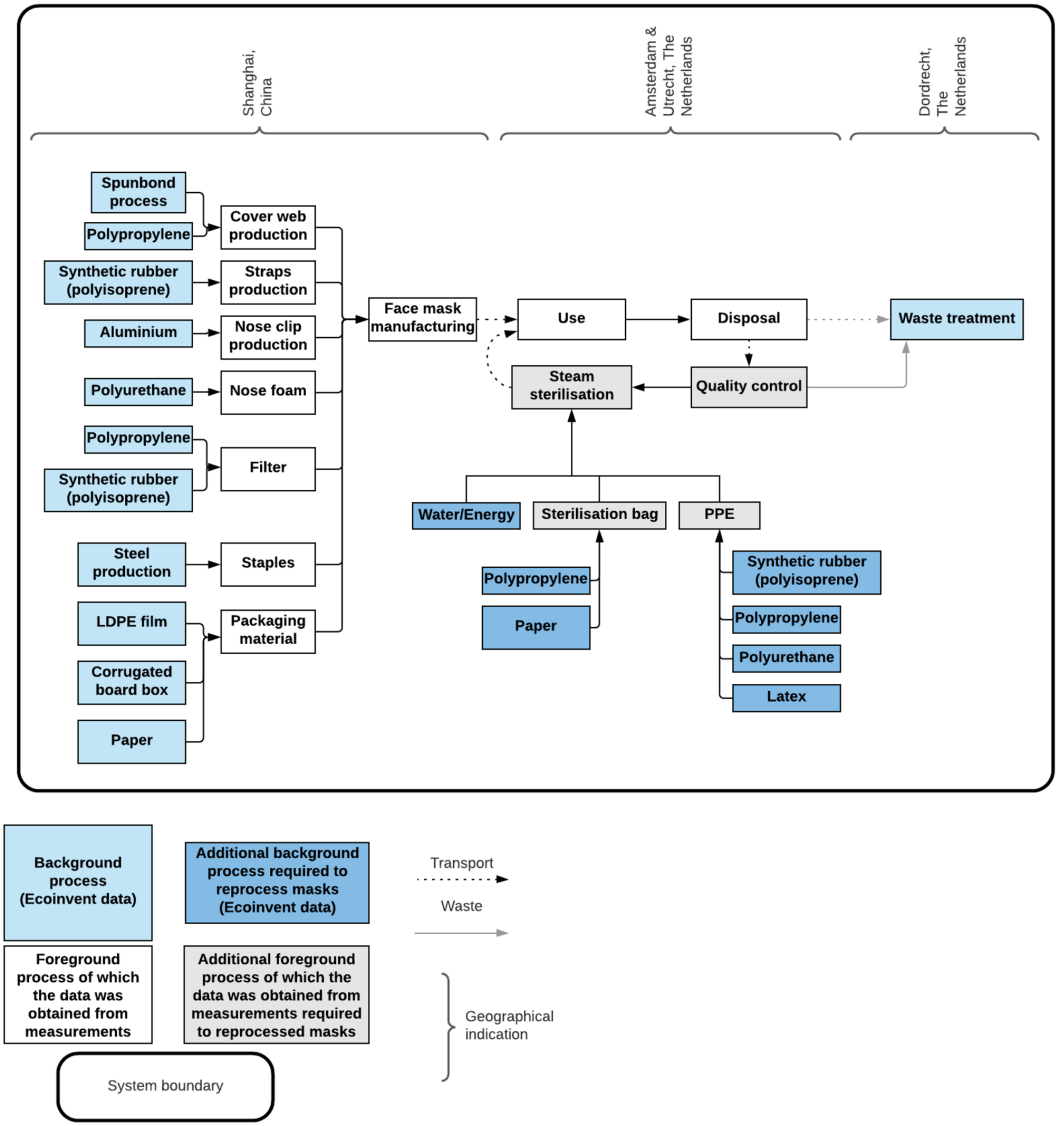
System boundary overview of new and reprocessed face masks including waste treatment by incineration.

To make a valid comparison between the disposable and reprocessing face masks, the system boundaries should be equal in both scenarios. The system boundaries in this study consisted of the production, the use and the disposal and waste treatment of the masks. For the reprocessed face masks, the lifecycle is extended due to the sterilisation process (Fig. 1). Therefore, the additional PPE’s and materials needed to safely process the masks (e.q. masks, gloves and protective sheets) are included in the production phase. The production of machinery for the manufacturing of the face masks and the autoclave were not included in this study.

The production facility for the face masks is located in Shanghai, China^22,23^. Further distribution took place from Bracknell, UK to Neuss, Germany and the final destination was set in Rotterdam, the Netherlands.

The packaging materials were disposed in the hospital where the face masks are used primarily. After first use, face masks were transported to the sterilisation department. All masks were manually checked before reprocessing by personnel wearing PPE. Of all used Aura 1862+ facemasks that entered the CSA, approximately 10% was discarded. To remain conservative, the LCA was conducted based on a 20% rejection rate as a result of face masks which could not be reused anymore due to deformities, lipstick, and broken elastic bands.

A full overview of the life cycle inventory table for the two scenarios and details on model assumptions are added in the Supplemental file (Supplemental file, Part B)*.*

### Life cycle impact assessment

The carbon footprint (kg CO_2_ eq) was chosen as the primary unit in the impact category. ReCiPe was applied at midpoint level and used to translate greenhouse gas emissions into climate change impact^16^.

### Uncertainty analysis

The final LCA model contains several uncertainties based on assumptions and measurement inaccuracies^24^. The included uncertainties were based on weighted components of the masks as well as the packaging which were measured with 1.5% inaccuracy of the precision scale apparatus. A Monte Carlo sampling^25^ was conducted for both alternatives (disposable and reprocessing) where input parameters for the LCA were sampled randomly from their respective statistical distributions in for 10,000 ‘runs’. Because input parameters between scenarios were partly overlapping, we compared these two scenarios directly using a discernibility analysis. This technique, establishes which scenario is beneficial for each of 10,000 Monte Carlo runs. We report the percentage of instances where the reprocessing scenario has a lower carbon footprint than the disposable scenario.

### Sensitivity analysis

A sensitivity analysis was conducted to check the sensitivity of the outcome measures to variation in the input parameters. To determine which parameters are interesting to investigate, three aspects were considered: the variations in number of face masks per sterilization cycle (autoclave capacity), rejection rate (number of losses per cycle) and transport distance to the CSSD. Finally, we included the relative contribution of these variations. The following three parameter variations were chosen for the sensitivity analysis:

1. Rejection percentage. The rejection rate was defined based on experiences from the participating sterilisation department and studies that show that sterilisation of the face masks up to 5 times is possible. Masks were re-used for 5 times, approximately 10% was discarded during the total life cycle. Out of this experience and to remain conservative, the total rejection rate was set on 20%. Therefore it is interesting to investigate whether variation in PFE testing outcomes or differences in user protocols influence the outcomes. This should indicate if masks from higher or lower quality can also be suitable candidates for reprocessing.
2. Autoclave capacity, which largely depends on the loading of the autoclave. To mimic different loads of the autoclave, it is interesting to know the influence of sterilizing fewer masks per run on the model.
3. Transport. As it is likely that many hospitals have a Central Sterilisation Services Department (CSSD) it is interesting to know the effect of having zero transportation. Moreover, in case hospitals are not willing to change the routing in their CSSD it is interesting to observe how outcomes are influenced if transportation is set on the maximal realistic value of 200 km.

The parameters have been varied with 250 and 500 face masks per sterilisation batch. A rate varying with 10% and 30% of the face masks being rejected due to quality reasons and variation in transport kilometres of 0–200 km.

There is a small difference between the baselines of the sensitivity, LCIA and contribution analyses because all these are performed using separate Monte-Carlo simulations. The output of the different simulations may show minor differences due to statistical distribution.

### Cost price comparison

A cost analysis was made to give insight in costing from a procurement perspective. The cost analysis is conducted with five face masks that were steam sterilized per batch in a permeable laminate bag, Halyard type CLFP150X300WI-S20 and includes the expenses of energy, depreciation, water consumption, cost of personnel, overhead and compared to the prices for a new disposable 3M Aura face mask during the first and second Corona waves. Five pieces per bag were chosen in order to have enough space between the masks to sterilise each mask properly. The cost analysis is based on actual sterilization as well as associated costs compared to the prices of new disposable face masks. The costs were then related to the functional unit of protecting 100 health care workers by calculating the difference in the amount of Euros per 100 face masks.

## Results

The impact category outcomes of new versus reprocessed face masks over a functional unit of 100 times use with Standard Deviation (SD) for carbon footprint are:

- 6.55E+00 (SD 3.11E−01) kg CO_2_ eq for new face masks.
- 2.77E+00 (SD 1.21E−01) kg CO_2_ eq for reprocessed face masks.

The carbon footprint is approximately 3 kg CO_2_ eq for 100 times of protection with a face mask which is (re)sterilized and reused for an additional five times.

The relative difference on carbon footprint is 58% lower for (re)sterilised face masks compared to face masks that are disposed after one time of use.

**Figure 2:**
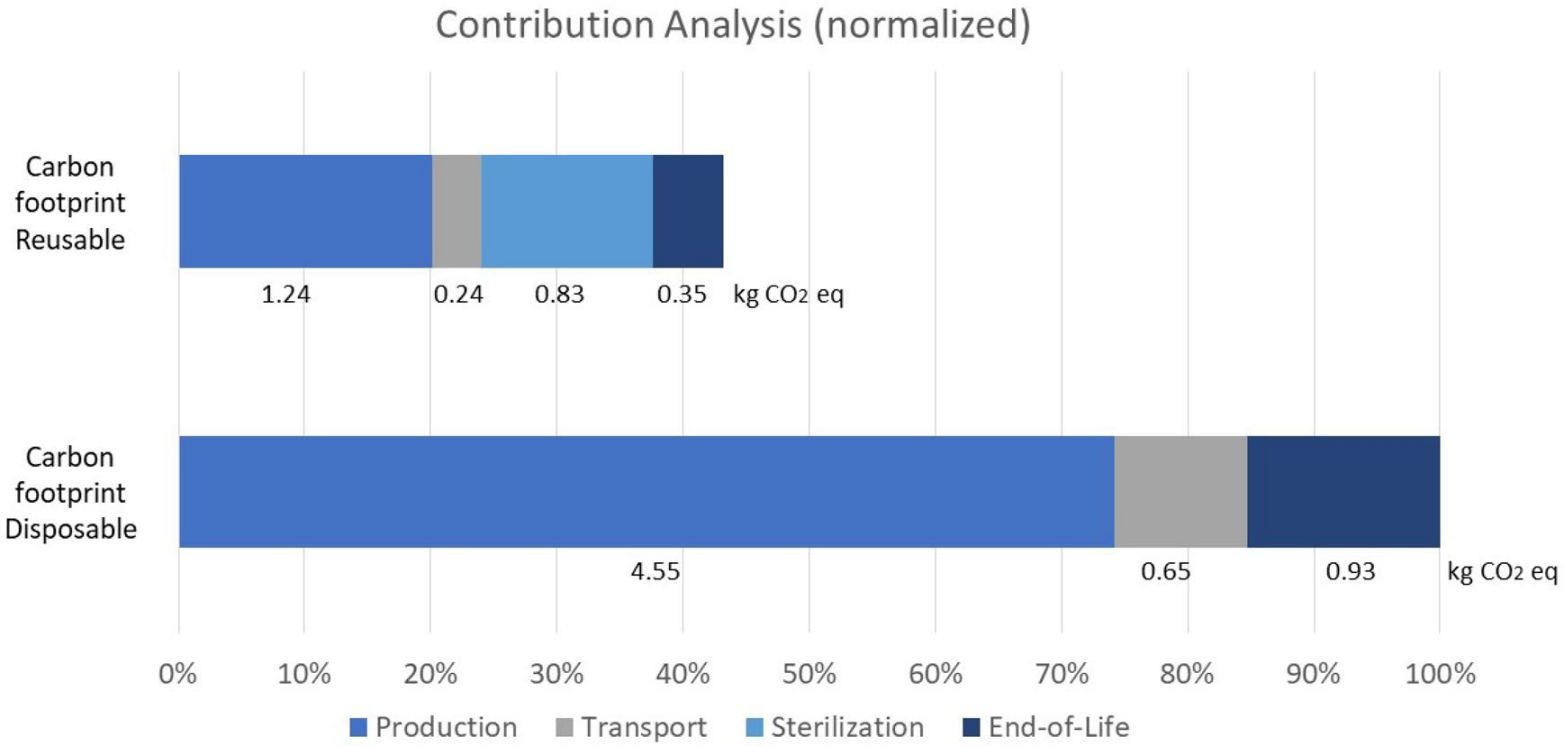
Sources of impact relative to the impact category carbon footprint.

Figure 2 shows the normalized contribution expressed in percentages of the total carbon footprint of a disposable mask.

Compared to disposal, reprocessing of face masks showed a lower climate change impact.

The contribution analysis shows that the highest reduction in climate change is caused by the production phase of the face masks. The production phase is reduced from 4.55 kg CO_2_ eq to 1.24 kg CO_2_ eq. Moreover, the impact caused by transport reduces from 0.65 to 0.24 kg CO_2_ eq and the end-of-life impact decreases from 0.93 to 0.35 kg CO_2_ eq.

Sterilization however, has a significant impact on the total results of reprocessed face masks. This phase contributes with 0.83 kg CO_2_ eq. When comparing to disposable masks, reprocessed face masks even when including sterilization, remains the alternative with a lower impact on climate change.

The discernibility analysis indicated that the reprocessing scenario had a lower climate change impact.

### Sensitivity analysis

**Figure 3:**
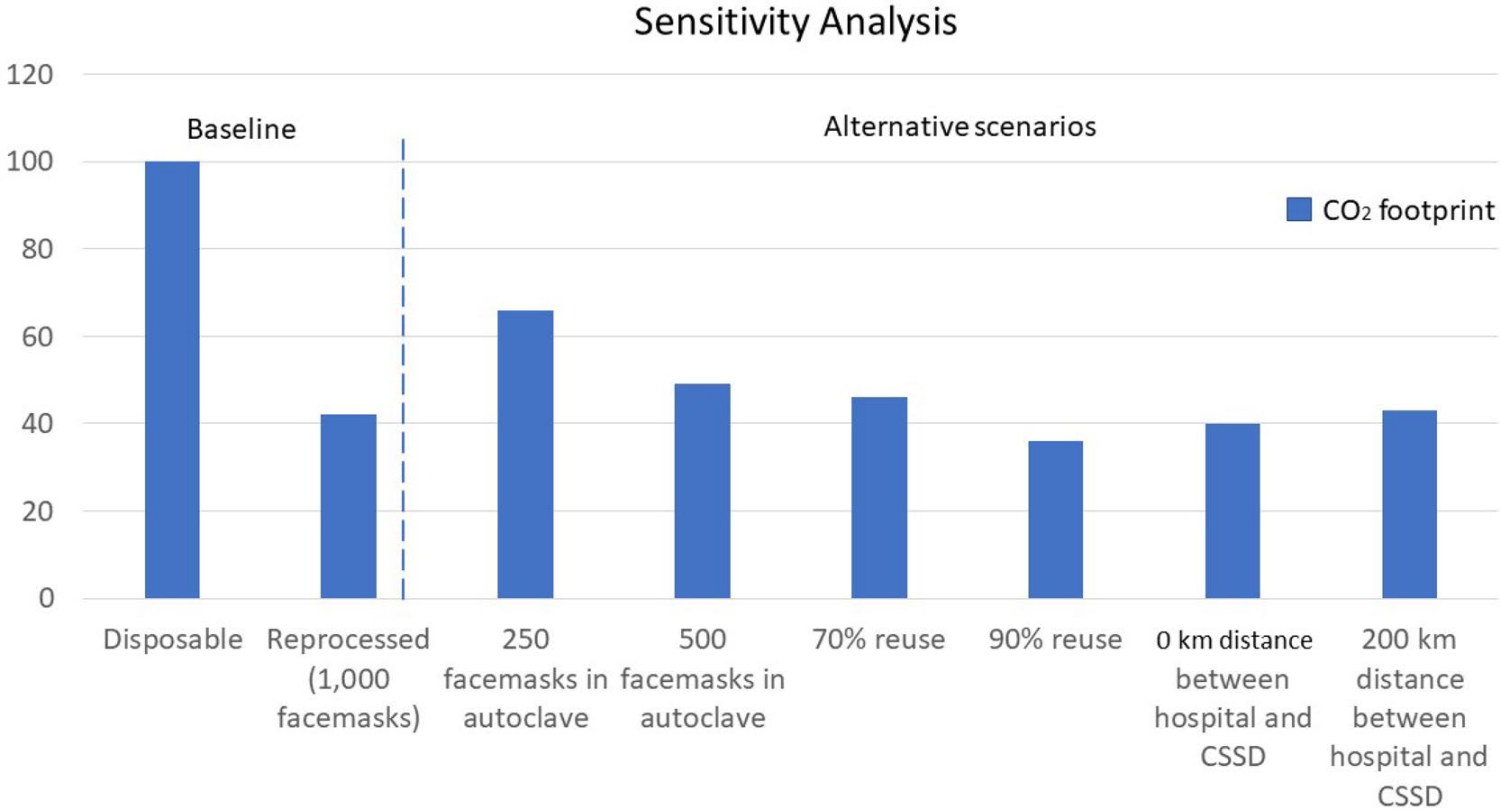
Sensitivity analysis. Left of dotted line, baseline disposable (masks used only one time) versus reprocessed face masks with benchmark autoclave loading capacity of 1000 masks, rejection rate of 20% and transport distance of 46.1 km. Right of dotted line, effect on carbon footprint of different autoclave loading capacity, different rejection rates and different transport distances from hospital to sterilisation site.

Figure 3 shows the sensitivity analysis outcomes, including the baseline results and results using different autoclave loading capacity, rejection rate after inspection and transport differences.

The baseline characteristics for the autoclave capacity was determined for 1000 face masks per cycle and a transport distance from the hospital to the CSSD of 46.1 km. We assumed that 80% of the masks were reused each time the mask came back into the CSSD for reprocessing. When the autoclave capacity drops to 250 or 500, the CO_2_ emissions increases by 57% and 17% respectively. If 70% of the face masks are reused, the CO_2_ emissions increase with 8%. If 90% of the face masks are reused, the emissions decrease with 14%. If the travel distance changes to 200 km as a max feasible distance, the CO_2_ emissions increases with 2%. Reducing the distance to 0 when the CSSD is inside the hospital decreases the emissions with 7%.

The results of the sensitivity analysis show that even with relatively large variations in changing parameters, the reprocessed face masks continue to have a lower impact in all categories when compared to using new disposable masks (Supplemental file, Part C). The uncertainty and discernibility analysis were performed (solely) over the weight of the mask. The results demonstrate that the inaccuracy of the scale does not affect the fact that reprocessed mask has a lower score in terms of climate change as compared to disposable masks.

### Cost price comparison

The cost price of a reprocessed mask by means of steam sterilization in a permeable laminate bag is €1.40. The purchase price of a new, disposable face mask was €7 during the peak time of shortages in the hospital Haaglanden MC. The prices after the first Corona wave dropped to €186.06 per 120, having an average price of €1.55 per Aura 1862 face mask. Saving 560 Euro per 100 protected healthcare workers in the first wave and 15 Euro in the second Corona wave when using reprocessed masks.

## Discussion and interpretation

The main finding in this study, when looking back at the research questions, demonstrate that there is a significant environmental benefit if FFP2 face masks are reprocessed. Therefore, reprocessing may contribute to achieving a circular economy as the Life Cycle Assessment indicates a 58% reduction in carbon footprint.

Furthermore, there is a price benefit when reprocessing face masks as compared to using new disposable masks. Although the prices after the first Corona wave dropped to €186.06 per 120, having an average price of €1.55 per Aura 1862 face mask for Haaglanden MC, this was still higher than the cost price of the reprocessed masks.

The sensitivity analysis conducted in this study indicates that both loading fewer face masks into an autoclave as well as variation in the rejection rate have a significant impact on the end result. The relative high impact of the sterilization process is in line with other studies that showed that the sterilization process is a critical process for the environmental footprint of sterile products^26^. Therefore, a hospital should take into consideration to optimally load an autoclave since the volume has a significant effect on the cost price and the climate change impact per product.

Next to steam sterilization, other methods of sterilization of face masks are available such as H_2_O_2_ plasma sterilization, Gamma radiation or UV irradiation sterilization^27^. However, Steam sterilization seems an attractive method as studies indicate better PFE results compared to H_2_O_2_ and gamma radiation^10^. Although steam sterilization is readily available at hospitals and because of the larger loading capacity, most autoclaves can handle much more face masks per cycle compared to most alternatives. However, it is interesting to perform an LCA based on those alternatives for comparison.

Disposable 3M 1862+ masks that were sterilized up to five times by means of steam sterilization at 121 °C showed good PFE results above 94% while maintaining their breathability and shape. However, other masks with similar FFP2 standard showed varying quality after sterilization^10^. Therefore, unknown masks should be tested after sterilization for PFE, pressure drop and facial fit before brought back into circulation. This should be taken into account when hospitals decide to reprocess different types or brands of face masks in times of shortage.

The LCA was conducted on the basis of a 20% rejection rate as a result of face masks which could not be reused anymore due to deformities, lipstick, and broken elastic bands. However, If better care would be taken for used face mask such as instructions on how to wear, store and treat masks properly, it is likely that the rejection percentage drops even further. This will result in a better outcome in number of masks to be reprocessed and therefore, further improving carbon footprint values.

When considering that hypothetically 200 million disposable face masks are used per year in the Netherlands in all healthcare institutions during the Corona period, an increase in carbon footprint is expected.﻿ Based on the outcome of this study we can conclude that reprocessing 200 million disposal face masks will lead to a reduction of 7.56 million kg CO_2_ eq (Supplemental file, Part D).

Many face masks that cannot be used anymore and need to be discarded were made of high-quality polypropylene material (Supplemental file, Part A) and can still contribute to the transition towards a circular economy for medical devices. Therefore, repurposing products at the end of their life cycle to be reused or to become raw material for new medical devices should be included in the design of these products. Then, modular design of masks could decrease the disassembly effort and therefore, reduce the climate change impact as well^28^.

This study focuses on the Life Cycle Assessment of disposed versus reprocessed face masks and may form a basis for further studies. These studies in the medical field can focus on instrument blue wrapping paper, drapes also made out of the Polypropylene material^6^ as well as anaesthetic and oxygen masks and oxygen tubing made out of other types of thermoplastic materials. These further studies should determine if they have similar environmental benefits. Reuse and reprocessing of medical products is possible such as with surgical instruments, especially when modular designed^29,30^.

Facing the climate challenges and following the EU’s 2020 climate goals it seems evident that the carbon footprint needs to be reduced. For policymakers it is important to realize that reducing the carbon footprint of medical devices such as face masks may have relevant positive effects on climate change. The insights from this study could help to reach the goals of the Green Deal^31^ by reducing the CO_2_ emissions by 49% in 2030.

## Limitations

Although it is likely that CSSD employees use a maximum autoclave loading, the LCA sensitivity analysis included variations in holding capacity. However, it remains more interesting to know the impact on the LCA if different types and sizes of autoclave are used that require different energy and water consumption. In order to better relate the model and study results to different CSSD setups (e.g. general practitioner, larger academic hospital etc.) a study expansion is needed that includes autoclave type in addition to holding capacity. The uncertainty analysis was only performed on the inaccuracy of the scale, weighing the masks. To improve the Monte Carlo analysis accuracy in further studies, it is advised to investigate the influence of all potential uncertainties, of foreground and background data.

Amongst other factors, the production of machinery for the manufacturing of the face masks and the autoclave are not included in this study since this data was not readily available, and therefore, outside the system boundary. Furthermore, the Global Inventory Data (GLO), representing the average global situation, and the data from the Rest of the World (RoW), average inventory data for all geographical areas not covered in ecoinvent, were combined in our study as not all data was available in the GLO dataset. Although not expected, further studies should identify if this may cause small deviations in outcome.

A Life Cycle Costing (LCC) has not been conducted in this study. An LCC would be recommendable in further studies in order to analyse the cost of each phase. Furthermore, it may be helpful for designers when designing new (circular) products.

The fit of the mask on the face of the users was determined by means of tactile and visual inspection. To ensure that masks remain fully functional and without damage after five reprocessing cycles it is advisable to check besides pressure drop and filter efficiency for inwards leakage with commercially available systems like the ACCUFIT system (AccuFIT 9000 Respirator Fit Test apparatus (https://accutec-ihs.com/accufit-9000) before reprocessing of face masks is implemented in any hospital.

## Conclusion

The results of this study showed clear benefits of reprocessing face masks. The LCA demonstrated a significantly lower climate change impact for reprocessed medical face masks compared to new. Furthermore, reprocessing results in lower costs. This study may serve as an inspiration for investigating the reprocessing of other medical devices due to the potential large climate change impact and cost reductions. Therefore, this study advocates that circular design engineering principles should be taken into account when designing medical devices. This will lead to more sustainable products that have a lower carbon footprint and may be manufactured at lower costs. A circular economy for medical devices may serve therefore, as potential to execute the goals of the Green Deal and the global sustainable development goals of the United Nations.

## Supplementary Information

Supplementary Information.
