# Supplementary material for: A life cycle assessment of reprocessing face masks during the Covid-19 pandemic
Source: Sci Rep. 2021 Sep 3;11:17680. doi: 10.1038/s41598-021-97188-5 (PMC8417283; doi:10.1038/s41598-021-97188-5)
Supplement: Supplementary file 1 — Supplementary Information. [file 41598_2021_97188_MOESM1_ESM.docx]

Supplemental file

**Supplementary information for: A life cycle assessment of reprocessing face masks during the COVID-19 pandemic**

**B. van Straten^1^, Sharina Ligtelijn^1^, Lieke Droog^2^, E. Putman^3^, J. Dankelman^1^, N.H. Sperna Weiland^4^, T. Horeman^1^**

*1. Delft University of Technology, Department of BioMechanical Engineering, Delft, the Netherlands*

*2. Delft University of Technology & Leiden University, Industrial Ecology, Delft, the Netherlands*

*3. VWS, Ministry of Health, Welfare and Sport (VWS), The Hague, the Netherlands*

*4. Amsterdam University Medical Center, Amsterdam, the Netherlands*

**Author for correspondence:**

Name: Bart van Straten
Department: Department of BioMechanical Engineering
Institution: Delft University of Technology
Address: Mekelweg 2, Building 34, 2628 CD Delft, The Netherlands.
Phone: +31(0)6-53 68 18 41
E-mail: b.j.vanstraten@tudelft.nl

Table of content

[Part A, Face mask material 3](#_Toc77251602)

[Part B, Inventory data 11](#_Toc77251603)

[Part C, LCIA Results 17](#_Toc77251604)

[Part D, Reduction of carbon emissions 18](#_Toc77251605)

# Part A, Face mask material

The 444 masks consisted of 101 different types of masks. From the 101 different types, the 3M 1862 and Kolmi Op-Air were tested the most on the test setup. The PFE results of 89 3M 1862 and 26 Kolmi Op-Air are provided in Table S1 for 0.3, 0.5, 1 and 5 µm particles. The results indicate that the 3M 1862 shows low PFE values after 2x Sterrad (H_2_O_2_ plasma) processing and Kolmi Op-Air shows low and inconsistant PFE values after 1x 121 ^o^C processing. Measurements conducted at Delft University of Technology, Department of Chemical Engineering.

**Table S1
Particle Filter Efficiency of two commonly used mask after either 121 ^0^C steam or H_2_O_2_ Plasma sterilisation**

| **Brand type** | **Number of masks** | **Sterilization method** | **0.3 µ**  **% PFE (SD)** | **0.5 µ**  **% PFE (SD)** | **1 µ**  **% PFE (SD)** | **5 µ**  **% PFE (SD)** | **Mean**  **% PFE** |
| --- | --- | --- | --- | --- | --- | --- | --- |
| **3M 1862** | 5 | H_2_O_2_  Sterrad | 86,4  (12,5) | 93,8  (6,2) | 97,4  (2,7) | 99,5  (0,5) | **94** |
| **3M 1862** | 72 | 121 ⁰C  steam | 93,6  (4,1) | 97,3  (2,1) | 99,0  (0,8) | 99,7  (0,7) | **97** |
| **3M 1862** | 4 | 2 x H_2_O_2_ Sterrad | 41,3  (1,7) | 66,9  (1,6) | 83,9  (1,3) | 99,5  (0,4) | **73** |
| **3M 1862** | 8 | 2 x 121 ⁰C steam | 91,6  (3,2) | 96,2  (1,8) | 98,3  (0,8) | 100  (0,1) | **97** |
| **Kolmi OP-Air M52010** | 11 | H_2_O_2_  Sterrad | 89,8  (1,4) | 96,4  (1,4) | 98,4  (0,5) | 99,8  (0,3) | **96** |
| **Kolmi OP-Air M52010** | 15 | 121 ⁰C  steam | 21,2  (6,8) | 56,3  (8,5) | 78,4  (8,2) | 99,8  (0,5) | **64** |

*Thermal properties of 3M Aura 1862+ and Kolmi Op-Air M52010 face masks using DSC*

The 5 different polymeric layers of each mask consisting of different texture, fiber size and orientation as macroscopically observable by naked eye, have individually undergone controlled thermal ramp (Figure 1).


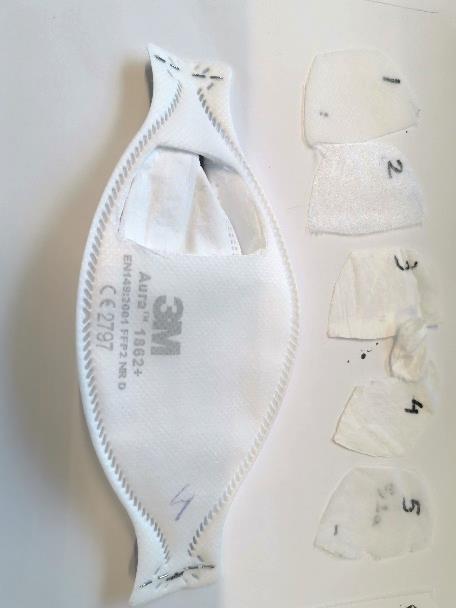


***Figure S1. Dissection of 5 constituent layers of 3M Aura 1862+ mask filter.***

The first heating traces of each constituent layers are shown in Figure S2. Upon heating of each layer, broad endothermic melting transition occurred over a larger temperature range. In the 3M Aura 1862+ mask filter (Figure S2a), the melting transition of the three first layers occurred over 130-168 ℃ while the endpoints of layer 4 and 5 shift to 174℃ and 180℃ respectively.

The melting transition peaks of the layers of Kolmi Op-Air M52010 masks varied in terms of the shape, and the melting occurred over 161-167 ℃ (Figure S2b). The enthalpy varied between 69 J/g for Layer 1 and 100 J/g for Layer 2 which indicate that these layers hold the same polymer composition or has a different fibre morphology, as the level of crystallinity is dependent on the polymer processing.

In order to eliminate the effect of fiber morphology and history of the samples, Layer 1 has undergone a second heating following a cooling ramp after first heating. As shown in Figure S3, the serrated shape of the melting transition has switched to a smooth melting peak which occurs at exactly the same temperature. This suggests that some of the DSC features in the first heating scan are due to (history dependent) artefacts, such as surface moisture, absorption of volatile substances. It is worth noting that normally DSC results on organic substances and polymers rely on using the second heating curve, as the first heating curve very frequently shows these artefacts. In the case of facemasks, and the actual use of the surface structure on the fibers in the mask material, we considered it useful to also show the first DSC heating curves. In the following, further complementary characterizations have been done in order to provide more detailed information over the physical and chemical composition of these layers.

***
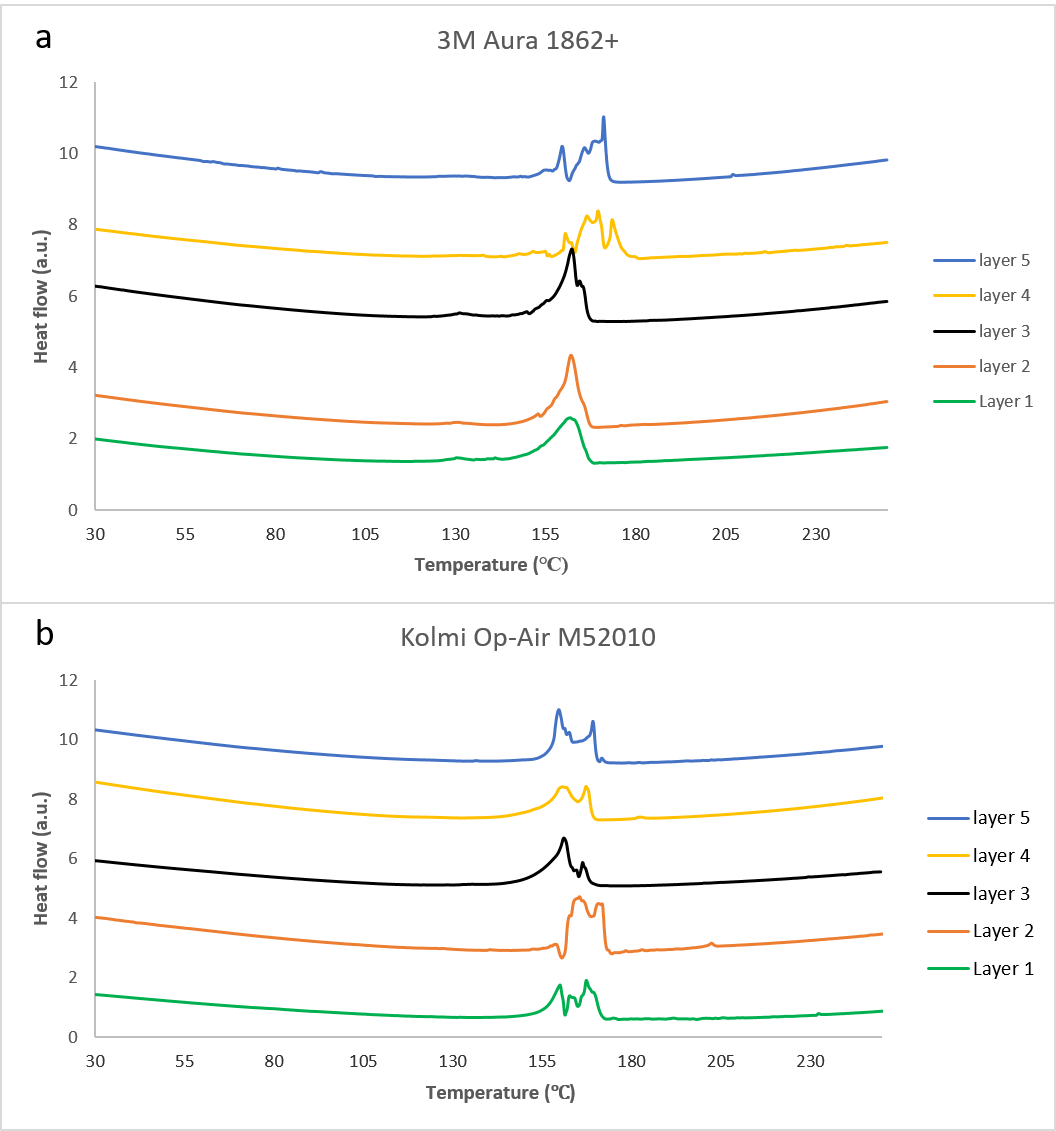
***

***Figure S2.* *DSC plots showing melting transitions of layers upon heating 10 degrees/min (endo up), a: 3M mask, b: Kolmi mask.***

*Figure S3. DSC traces (heat-cool- heat cycles under 10℃/min) on layer 1 of 3M Aura 1862+ mask filter.*

*XRD measurements*

The Bruker D8 X-Ray Diffraction test results (Figure S4) for the 3M Aura 1862+ mask reveal 4 peaks for the 3M mask layers 1,2,3 and Kolmi 1,3,4 peaks at 16, 20, 22 and 25 degrees and 16, 20, 22 and 25 degrees. For the layers 4 and 5 of the 3M mask, peaks were found at 16, 20 and 22 degree. For the layers 2 and 5 of the Kolmi mask, peaks were also found at 16, 20 and 22 degrees. Note that small shifts are probably caused by sample surface heights. Furthermore, it was observed that the amplitude expressed in counts was higher for all layers of the 3M masks compared to the Kolmi.


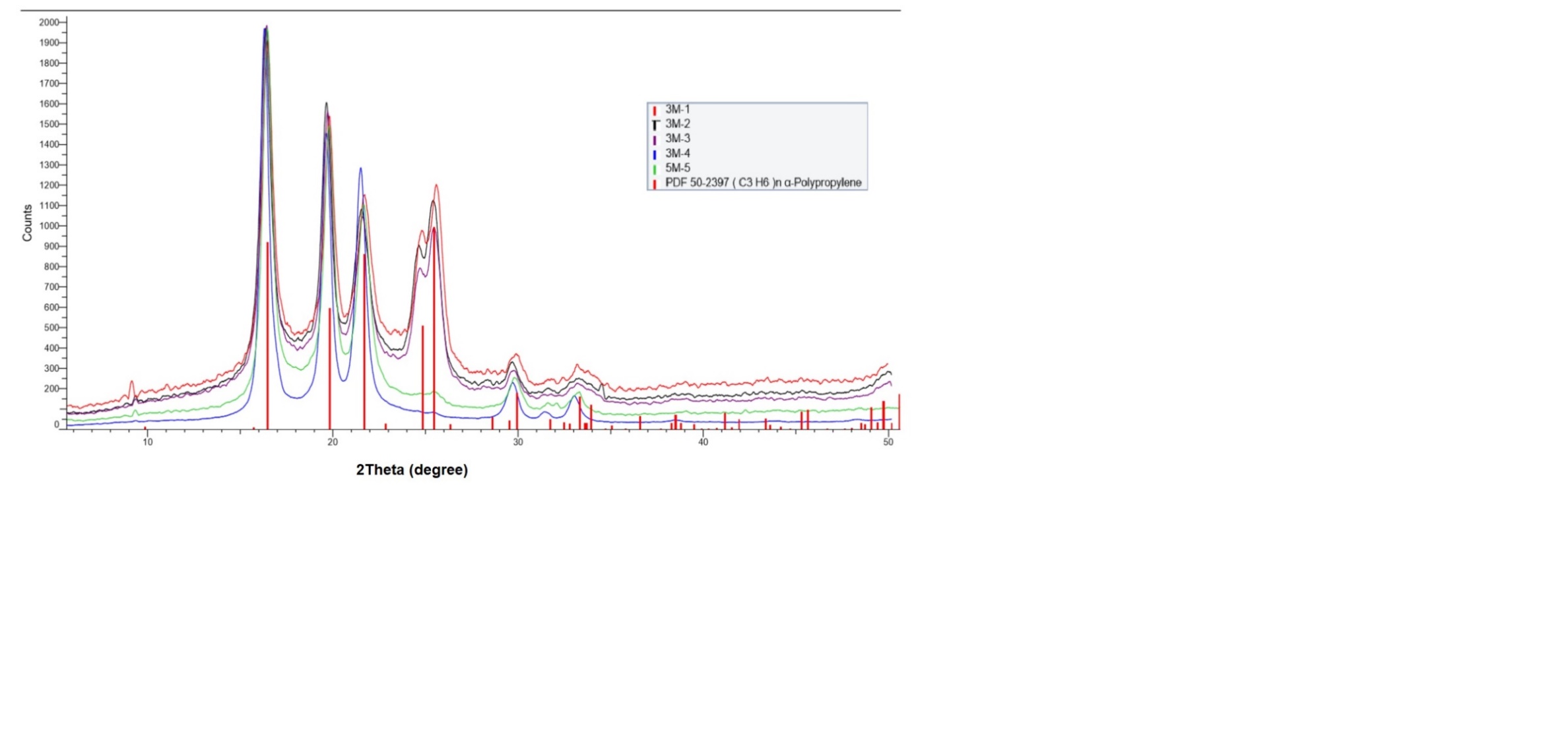


**
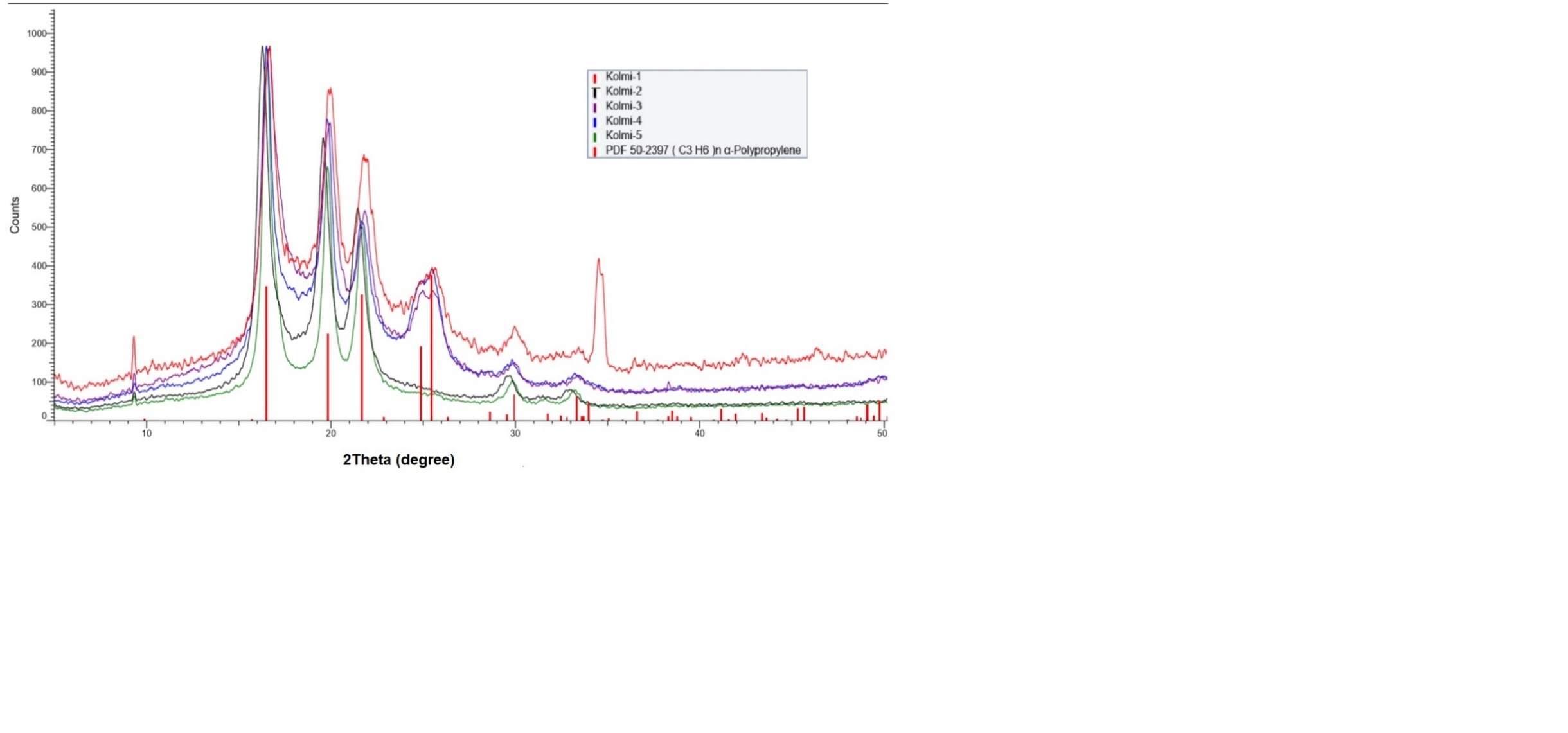
**

***Figure S4. The Bruker D8 X-Ray Diffraction test results with Polypropylene reference data for 3M (top) and***

***Kolmi (bottom).***

Upper, XRD pattern of the 5 layers of 3M 1862+ after normalization to max intensity. Below, XRD pattern the layers of Kolmi Op-Air after normalization to max intensity.

*Infrared Spectroscopy (FTIR)*

The test results from the Infrared Spectroscopy confirmed a match of all 5 layers of both masks with the profile of the material PP. Figure 5 shows the comparison between layer 1 from 3M, layer 2 from Kolmi.

**

***Figure S5. The FTIR spectra of layer 1 from 3M mask (Top), and layer 2 from Kolmi mask (Bottom).***

Characteristics peaks of PP are indicated by grey lines. The functional group assignment and vibration type of each PP peak can be found at Fang et al. (2012). Figure S6 presents the FTIR spectra graphs of all 5 layers and characteristics peaks of PP.


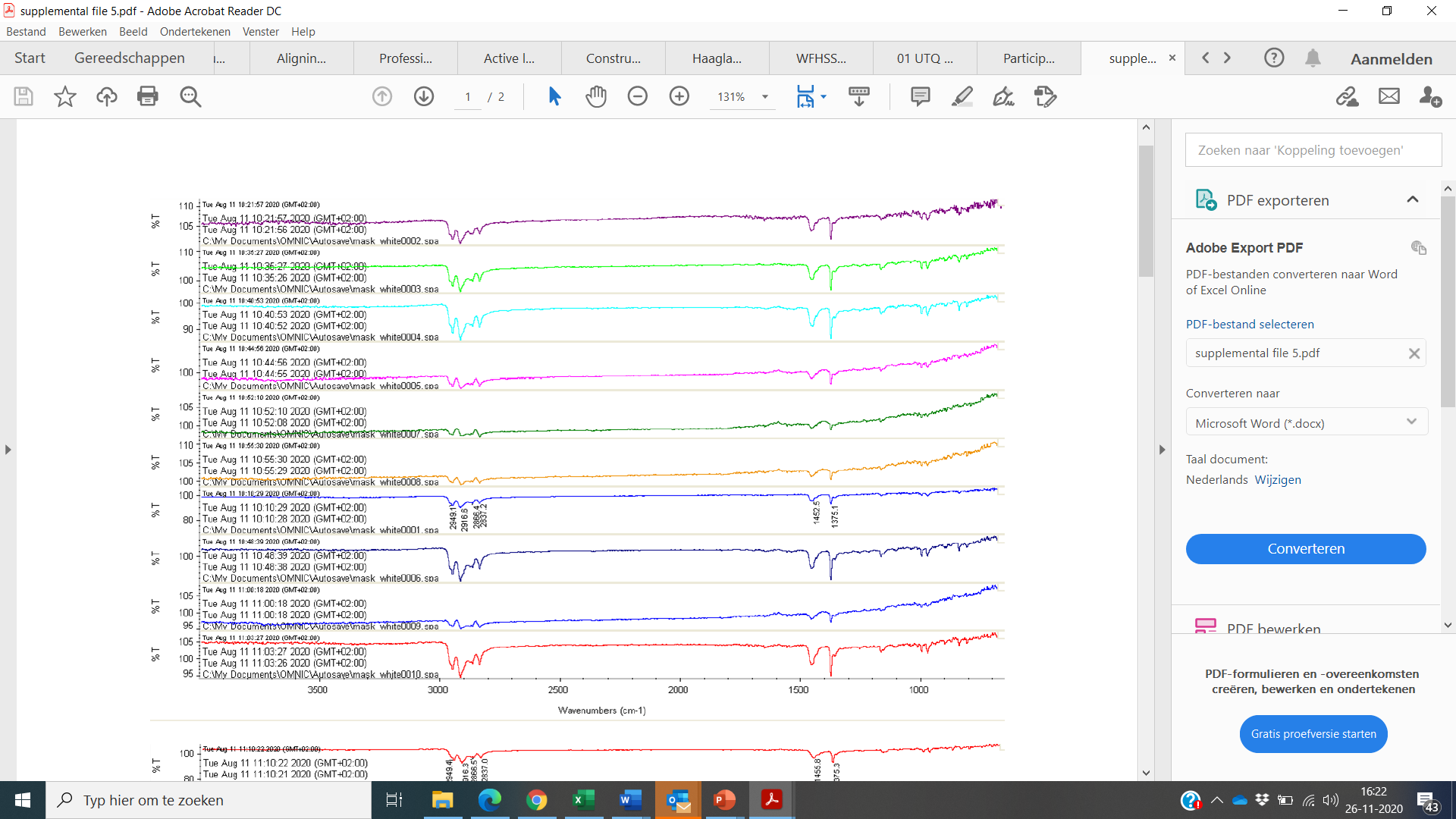


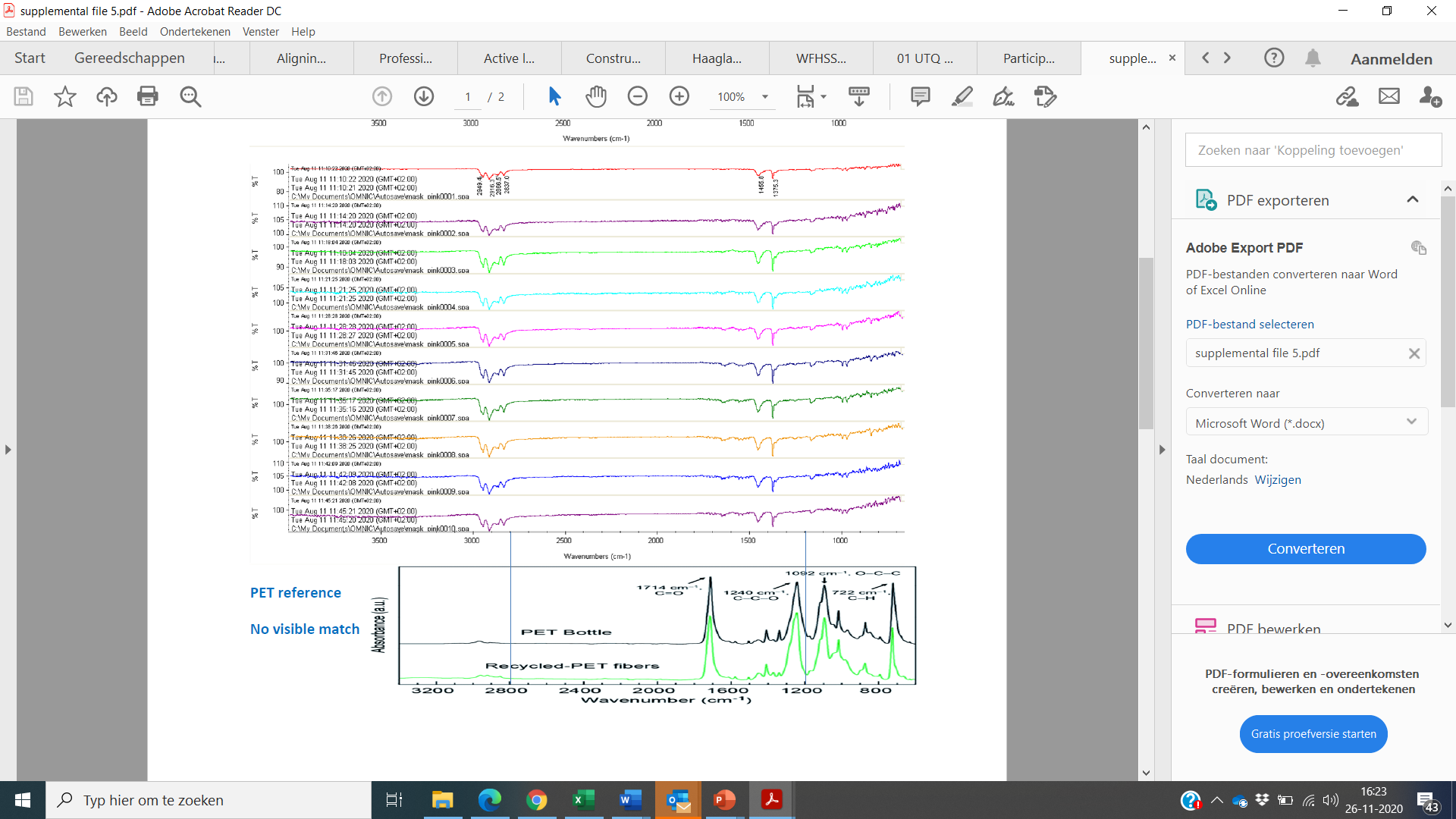


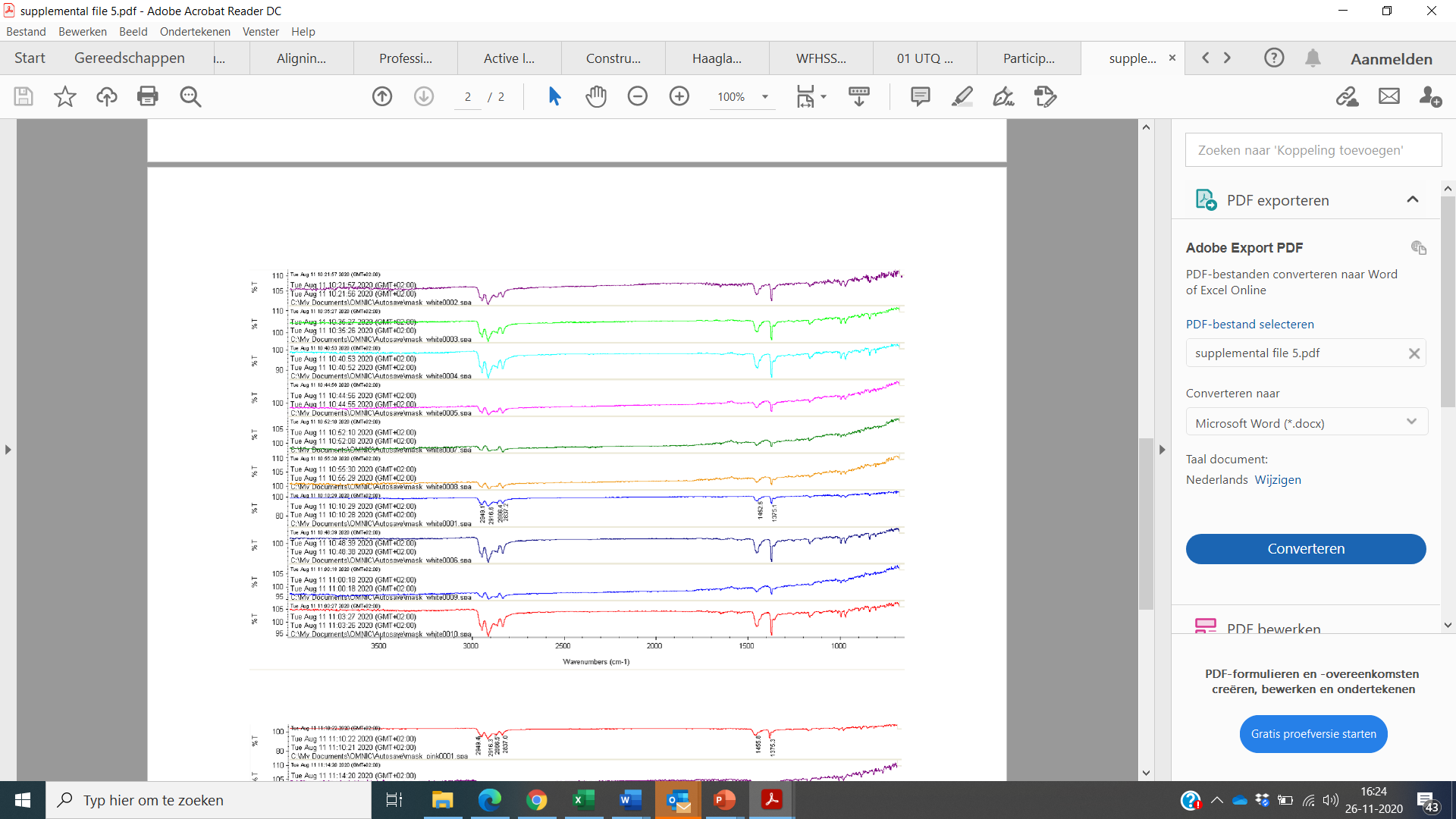


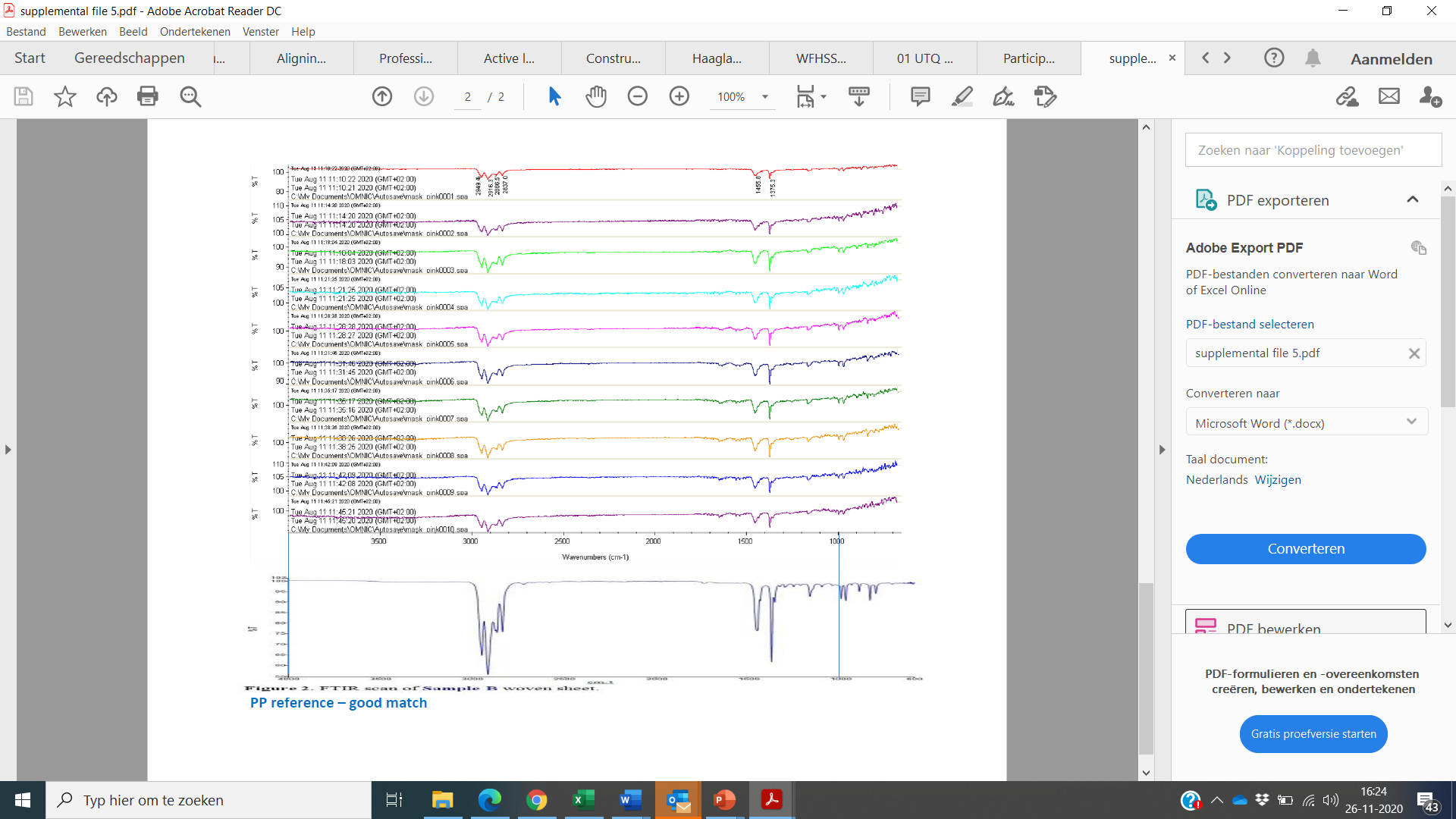


***Figure S6 the FTIR spectra graphs of all 5 layers and characteristics peaks of PP***

# Part B, Inventory data

***Table S2, Alternative 1 single use of face mask***

| **Production of 3M Aura Face Mask** | | | |
| --- | --- | --- | --- |
| **Economic inputs** | | | |
| **Product** | | **Amount** | **Ecoinvent name** |
| 1 | Fabric face mask | 5.82 gram | Textile, non-woven polypropylene {RoW}\| textile production, non woven polypropylene, spun bond \| Cut-off, S |
| 2 | Elastic straps | 0.97 gram | Synthetic rubber {GLO}\| market for \| Cut-off, S |
| 3 | Nose guard | 0.61 gram | Polyurethane, flexible foam {RoW}\| market for polyurethane, flexible foam \| Cut-off, S |
| 4 | Staples | 0.48 gram | Steel, chromium steel 18/8 {GLO}\| market for \| Cut-off, S |
| 5 | Nose clip | 0.72 gram | Aluminium, wrought alloy {GLO}\| market for \| Cut-off, S |
| 6 | Plastic wrap around single mask | 1.22 gram | Extrusion of plastic sheets and thermoforming, inline {GLO}\| market for \| Cut-off, S |
| 7 | Manual | 0.45 gram | Printed paper {GLO}\| market for \| Cut-off, S |
| 8 | Small cardboard box | 3.91 gram | Corrugated board box {RoW}\| production \| Cut-off, S |
| 9 | Large cardboard box | 2.36 gram | Corrugated board box {RoW}\| production \| Cut-off, S |
| 10 | Plastic wrap around large cardboard box | 1.39 gram | Packaging film, low density polyethylene {GLO}\| market for \| Cut-off, S |
| 11 | Transport from factory to port Shanghai | 0.00032 TKM | Transport, freight, lorry 16-32 metric ton, euro5 {RER}\| market for transport, freight, lorry 16-32 metric ton, EURO5 \| Cut-off, S |
| 12 | Transport from port Shanghai to port Southampton | 0.389 TKM | Transport, freight, sea, container ship {GLO}\| market for transport, freight, sea, container ship \| Cut-off, S |
| 13 | Transport to port Southampton to end-user | 0.0167 TKM | Transport, freight, lorry 16-32 metric ton, euro4 {RoW}\| market for transport, freight, lorry 16-32 metric ton, EURO4 \| Cut-off, S |
| **Environmental inputs** | | | |
| **Product** | | **Amount** | **Ecoinvent name** |
|  | N/A |  |  |
| **Economic outputs** | | | |
| **Product** | | **Amount** | **Ecoinvent name** |
|  | 3M Aura Facemask | 1 piece |  |
| 14 | Disposal of plastic wrap | 1.22 gram | Municipal solid waste {NL}\| market for municipal solid waste \| Cut-off, S |
| 15 | Disposal of manual | 0.45 gram | Waste graphical paper {NL}\| market for waste graphical paper \| Cut-off, S |
| 16 | Disposal of small cardboard box | 3.91 gram | Waste paperboard {NL}\| market for waste paperboard \| Cut-off, S |
| 17 | Disposal of large cardboard box | 2.36 gram | Waste paperboard {NL}\| market for waste paperboard \| Cut-off, S |
| 18 | Disposal of plastic wrap carboard box | 1.39 gram | Waste plastic, mixture {NL}\| market for waste plastic, mixture \| Cut-off, S |
| 19 | Disposal of face mask | 8.57 gram | Municipal solid waste {NL}\| market for municipal solid waste \| Cut-off, S |
| **Environmental outputs** | | | |
| **Product** | | **Amount** | **Ecoinvent name** |
|  | N/A |  |  |

1 – 5:
The facemask was disassembled and each part was weighted with a precision scale (Fit Evolve, Bangosa Digital, Groningen, the Netherlands) with a calibrated inaccuracy of 1.5%. Based on the technical data sheet provided by the manufacturer, the main material of each component was determined.

6 – 10:
The packaging material was weighted with a precision scale (Fit Evolve, Bangosa Digital, Groningen, the Netherlands) with a calibrated inaccuracy of 1.5%. Based on the technical data sheet provided by the manufacturer, the main material of each component was determined.

11:
3M masks are produced in Shanghai, China. The distance between the production location of 3M in Shanghai and the industrial port is estimated on 15.9 km. The weight of the facemask and packaging is 17.93 gram. Hence, the transport between the factory and port is 0.00032 TKM per face mask.

12:
3M masks are transported from Shanghai, China to Southampton, United Kingdom. The distance between the two ports is estimated on 21694.33 km. The weight of the facemask and packaging is 17.93 gram. Hence, the transport between the factory and port is 0.38898 TKM per face mask.

13:
From the port in Southampton, the masks are transported to Bracknell, United Kingdom, Neuss, Germany and finally, the end-user is assumed to be located in Rotterdam, The Netherlands. This results in a total distance of 932.77 KM. With a weight of 17.93 gram, the total transport was 0.0167 TKM per facemask.

14 – 18:
The disposal is based on the weight and the traditional waste treatment scenarios. The manual of 9.33 gram is divided over the 20 facemasks in which box it is included. The small cardboard box of 77.09 is divided over the 20 facemasks that are transported in the box and finally the large cardboard box of 150.3 grams is divided over the total 120 facemasks that are stored in the box.

***Table S3, Alternative 2 reprocessing of face mask***

| **Production of Personal Protection Equipment (PPE)** | | | |
| --- | --- | --- | --- |
| **Economic inputs** | | | |
| **Product** | | **Amount** | **Ecoinvent name** |
| 1 | Single use 3M Aura face mask | 1 piece | N/A (see alternative 1) |
| 2 | Protective suit | 133.04 gram | Textile, non-woven polypropylene {GLO}\| market for textile, non woven polypropylene \| Cut-off, S |
| 3 | Packaging of protective suit | 5.04 gram | Packaging film, low density polyethylene {GLO}\| market for \| Cut-off, S |
| 4 | drape | 102.51 gram | Textile, non-woven polypropylene {GLO}\| market for textile, non woven polypropylene \| Cut-off, S |
| 5 | Hair cover | 2.22 gram | Textile, non-woven polypropylene {GLO}\| market for textile, non woven polypropylene \| Cut-off, S |
| 6 | Pair of gloves | 16.71 gram | Latex {RER}\| market for latex \| Cut-off, S |
| 7 | Goggles (main part) | 23.44 gram | Polyethylene terephthalate, granulate, amorphous {GLO}\| market for \| Cut-off, S |
| 8 | PUR foam (goggles) | 6.5 gram | Polyurethane, flexible foam {RoW}\| market for polyurethane, flexible foam \| Cut-off, S |
| 9 | Elastics straps (goggles) | 1.34 gram | Synthetic rubber {GLO}\| market for \| Cut-off, S |
| **Environmental inputs** | | | |
| **Product** | | **Amount** | **Ecoinvent name** |
|  | N/A |  |  |
| **Economic outputs** | | | |
| **Product** | | **Amount** | **Ecoinvent name** |
|  | Set of PPE | 1 set |  |
| 10 | Disposal of PPE | 299 gram | Municipal solid waste {NL}\| market for municipal solid waste \| Cut-off, S |
| **Environmental outputs** | | | |
| **Product** | | **Amount** | **Ecoinvent name** |
|  | N/A |  |  |

1:
A set of Personal Protection Equipment includes a single use face mask, such as the 3M aura

2 – 9:
Each component of a set of Personal Protection Equipment was weighted using a precision scale (Fit Evolve, Bangosa Digital, Groningen, the Netherlands) with a calibrated inaccuracy of 1.5%. The main material of each component was determined by using declarations on the website of a manufacturer of a similar product.

10:
The disposal is based on the weight of the total set of Personal Protection Equipment and a traditional waste treatment process in the Netherlands.

| **Production of a sterilization bag** | | | |
| --- | --- | --- | --- |
| **Economic inputs** | | | |
| **Product** | | **Amount** | **Ecoinvent name** |
| 1 | Lower part of the sterilization bag | 5.85 gram | Kraft paper, bleached {GLO}\| market for \| Cut-off, S |
| 2 | Upper part of the sterilization bag | 4.37 gram | Polyethylene, high density, granulate {GLO}\| market for \| Cut-off, S |
| **Environmental inputs** | | | |
| **Product** | | **Amount** | **Ecoinvent name** |
|  | N/A |  |  |
| **Economic outputs** | | | |
| **Product** | | **Amount** | **Ecoinvent name** |
|  | Sterilization bag | 1 piece |  |
| 3 | Disposal of sterilization bag | 10.22 gram | Municipal solid waste {NL}\| market for municipal solid waste \| Cut-off, S |
| **Environmental outputs** | | | |
| **Product** | | **Amount** | **Ecoinvent name** |
|  | N/A |  |  |

***Table S4, production of sterilisation bag for alternative 2 reprocessing of face mask***

1 & 2:
Each part of a set of sterilization bag was weighted using a precision scale (Fit Evolve, Bangosa Digital, Groningen, the Netherlands) with a calibrated inaccuracy of 1.5%. The main material of each component was determined by using declarations on the website of a manufacturer of a similar product.

3:
The disposal is based on the weight of the total set of Personal Protection Equipment and a traditional waste treatment process in the Netherlands.

| **Reprocessing of facemask** | | | |
| --- | --- | --- | --- |
| **Economic inputs** | | | |
| **Product** | | **Amount** | **Ecoinvent name** |
| 1 | Set of PPE | 0.0005 piece | N/A |
| 2 | Sterilization bag | 0.2 piece | N/A |
| 3 | Tap water | 0.228 liter | Tap water {Europe without Switzerland}\| market for \| Cut-off, S |
| 4 | Electricity | 0.0109 kWh | Electricity, low voltage {NL}\| market for \| Cut-off, S |
| 5 | Transport between hospital and sterilization site | 0.00084 TKM | Transport, freight, lorry 16-32 metric ton, euro5 {RER}\| market for transport, freight, lorry 16-32 metric ton, EURO5 \| Cut-off, S |
| **Environmental inputs** | | | |
| **Product** | | **Amount** | **Ecoinvent name** |
|  | N/A |  |  |
| **Economic outputs** | | | |
| **Product** | | **Amount** | **Ecoinvent name** |
| 6 | Quality control, rejection rate | 0.2 piece | Municipal solid waste {NL}\| market for municipal solid waste \| Cut-off, S |
| 7 | Sterilized face mask | 1 piece |  |
| 8 | Waste water | 0.228 kg | Wastewater, unpolluted {RoW}\| treatment of, capacity 5E9l/year \| Cut-off, S |
| **Environmental outputs** | | | |
| **Product** | | **Amount** | **Ecoinvent name** |
|  | N/A |  |  |

***Table S5, meta data reprocessing of face mask***

1:
Two autoclaves with a capacity of 1000 facemasks and a cycle of 1 hour are operated by two persons using a set of PPE each for two hours. Hence, the input of PPE is $\frac{2}{(1000 \times2 \times2)} = 0.0005$ piece.

2:
Each sterilization bag can be used to sterilize 5 facemask. Subsequently, the input of a sterilization is 0.2 piece.

3:
Per cycle, the autoclave uses 228 liter tap water. This means that, with a capacity of 1000 face masks for each cycle, a total amount of 0.228 liter can be accounted to each sterilized mask.

4:
Per cycle, the autoclave uses 10.9 kWh electricity. This means that, with a capacity of 1000 face masks for each cycle, a total amount of 0.0109 kWh can be accounted to each sterilized mask.

5:
The transport between the hospital in Rotterdam and the sterilization site was estimated on 49.1 km. Each sterilization cycle, the face masks were transported to and from the sterilization site. With a weight of 8.57 gram, the total transport was 0.00084 TKM.

6:
During quality control the rejection rate was 20% so 0.2 piece was disposed as municipal waste.

7:
The output of the sterilization process is 1 sterilized facemasks.

8:
The input of tap water is disposed to the waste water treatment system.

# Part C, LCIA Results

| Project | 3M-Aura_Reprocessing | |  |
| --- | --- | --- | --- |
| Calculation: | Compare |  |  |
| Results: | Impact assessment | |  |
| Product 1: | 1 p LCA mask reprocessing (of project 3M-Aura_Reprocessing) | | |
| Method: | ReCiPe 2016 Midpoint (H) V1.04 / World (2010) H | | |
| Indicator: | Characterization | |  |
| Skip categories: | Never |  |  |
| processes: | No |  |  |
| Exclude long-term emissions: | No |  |  |
| Sorted on item: | Impact category |  |  |

***Table S6, Output of the different simulations show minor differences due to statistical spread and differences due to multiple simulations of the same datasets. Raw data output:***

| **LCIA results** | **Impact** |  | **Unit** | **Mean for new face masks** |  | **Mean for reprocessed masks** |  | **Difference** |
| --- | --- | --- | --- | --- | --- | --- | --- | --- |
|  | Global warming |  | kg CO2 eq | 6,55E+00  (SD 3,11E-01) |  | 2,77E+00  (SD 1,21E-01 ) |  | 58% |
|  |  |  |  |  |  |  |  |  |
| **Sensitivity reprocessed** |  |  |  |  |  |  |  |  |
| **Impact category** | **Unit** | **Original Scenario** | **Autoclave 250st** | **Autoclave 500st** | **Reuse 70%** | **Reuse  90%** | **Transport  0 km** | **Transport 200 km** |
| Global warming  (carbon foorprint) | kg CO2 eq | 2.6494 | 4.3533 | 3.2174 | 2.9815 | 2.3719 | 2.5897 | 2.8329 |
|  |  |  |  |  |  |  |  |  |
| **Uncertainty reprocessed** |  |  |  |  |  |  |  |  |
| **Impact category** | **Unit** | **Mean** | **Median** | **SD** | **CV** | **2,5%** | **97,5%** | **SEM** |
| Global warming  (carbon foorprint) | kg CO2 eq | 2.7668 | 2.7646 | 0.1209 | 4.3712 | 2.5414 | 3.0109 | 0.0012 |
| **Uncertainty disposable** |  |  |  |  |  |  |  |  |
| Global warming  (carbon foorprint) | kg CO2 eq | 6.5544 | 6.5536 | 0.3110 | 4.7454 | 5.9932 | 7.1503 | 0.0031 |
|  |  |  |  |  |  |  |  |  |
| **Contribution reprocessed** |  |  |  |  |  |  |  |  |
| **Impact category** | **Unit** | **Total** | **Top** | **Mask production** | **Transport** | **Waste** | **Packaging** | **Sterilization** |
| Global warming  (carbon foorprint) | kg CO2 eq | 2.6494 | 0.0000 | 0.9399 | 0.2354 | 0.3470 | 0.2963 | 0.8309 |
| **Contribution disposable** |  |  |  |  |  |  |  |  |
| Global warming  (carbon foorprint) | kg CO2 eq | 6.1275 | 0.0000 | 3.4573 | 0.6472 | 0.9333 | 1.0896 |  |

| **Impact category** | **A >= B** | **Mean** | **Median** | **SD** | **CV** | **25%** | **75%** | **SEM** |
| --- | --- | --- | --- | --- | --- | --- | --- | --- |
| Global warming | 100 | 3.79402 | 3.79074 | 0.236035 | 6.221236 | 3.615896 | 3.966964 | 0.00236 |

# Part D, Reduction of carbon emissions

Reduction of 7.56 million kg CO2 eq which could be achieved when reprocessing 200 million face masks:

6.55 – 2.77 kg CO_2_ eq / 100 * 200 million = 7.56 million kg CO2 eq.
